# New York

**Published:** 1896-07

**Authors:** 


*New York.*—The master shoers of New York have succeeded in having their bill passed through the State Legislature and signed by the Governor. Already the New York County Veterinary Medical Association has recommended Dr. H. D. Gill as one of the Board, and his appointment has been favorably indorsed by the Master Shoers of New York County. This is the first bill of the kind engrafted upon the statutes of any of the States, and much good is expected from it in the way of better educated master shoers.

Dr. Gill is one of the editors of the JOURNAL, and is specially well equipped to fill the rôle of examiner.

